# The effects of industry funding and positive outcomes in the interpretation of clinical trial results: a randomized trial among Dutch psychiatrists

**DOI:** 10.1186/s12910-019-0405-7

**Published:** 2019-09-18

**Authors:** Joeri K. Tijdink, Yvo M. Smulders, Lex M. Bouter, Christiaan H. Vinkers

**Affiliations:** 1 Department of Philosophy, Faculty of Humanities, VU Vrije Universiteit, 1081 HZ Amsterdam, Netherlands; 2 Department of Medical Humanities, Amsterdam UMC, (location VUmc), Amsterdam, the Netherlands; 3 Department of Internal Medicine, Amsterdam UMC, (location VUmc), 1081 HZ Amsterdam, the Netherlands; 4 Department of Epidemiology & Biostatistics, Amsterdam UMC (location VUmc), 1081 HZ Amsterdam, the Netherlands; 5 Department of Psychiatry, and Department of Anatomy and Neurosciences, Amsterdam UMC (location VUmc), Amsterdam, the Netherlands

**Keywords:** Industry funding, Conflict of interest, Positive outcome bias, Credibility, Industry funding disclosure, Psychiatrists, Methodological quality, Perceived credibility, Perceived clinical relevance

## Abstract

**Background:**

Most studies are inclined to report positive rather than negative or inconclusive results. It is currently unknown how clinicians appraise the results of a randomized clinical trial. For example, how does the study funding source influence the appraisal of an RCT, and do positive findings influence perceived credibility and clinical relevance? This study investigates whether psychiatrists’ appraisal of a scientific abstract is influenced by industry funding disclosures and a positive outcome.

**Methods:**

Dutch psychiatrists were randomized to evaluate a scientific abstract describing a fictitious RCT for a novel antipsychotic drug. Four different abstracts were created reporting either absence or presence of industry funding disclosure as well as a positive or a negative outcome. Primary outcomes were the perceived credibility and clinical relevance of the study results (10-point Likert scale). Secondary outcomes were the assessment of methodological quality and interest in reading the full article.

**Results:**

Three hundred ninety-five psychiatrists completed the survey (completion rate 45%). Industry funding disclosure was found not to influence perceived credibility (Mean Difference MD 0.12; 95% CI − 0.28 to 0.47, p?) nor interpretation of its clinical relevance (MD 0.14; 95% CI − 0.54 to 0.27, p?). A negative outcome was perceived as more credible than a positive outcome (MD 0.81 points; 95% Confidence Interval (CI) 0.43 to 1.18, p?), but did not affect clinical relevance scores (MD -0.14; 95% CI − 0.54 to 0.27).

**Conclusions:**

In this study, industry funding disclosure was not associated with the perceived credibility nor judgement of clinical relevance of a fictional RCT by psychiatrists. Positive study outcomes were found to be less credible compared to negative outcomes, but industry funding had no significant effects. Psychiatrists may underestimate the influence of funding sources on research results. The fact that physicians indicated negative outcomes to be more credible may point to more awareness of existing publication bias in the scientific literature.

**Electronic supplementary material:**

The online version of this article (10.1186/s12910-019-0405-7) contains supplementary material, which is available to authorized users.

## Background

Several factors are known to influence the interpretation of research findings by editors, reviewers, and scientists [1–4]. Of these factors, study outcome and funding disclosure are important [2, 5]. The excess of positive results in the scientific literature, most likely due to selective reporting, is widely acknowledged [6, 7]. In addition, it has been firmly established that industry sponsored studies more frequently report positive results when compared to non-sponsored trials [8, 9], and that ‘negative’ industry-sponsored studies remain unpublished [10–13]. However, a limited number of studies have addressed factors that influence physicians in their interpretation of the scientific literature. This topic is important since physicians often fail to recognize the impact of conflicts of interest [14], even though a critical attitude by medical doctors towards industry funding has been found to affect the perceived relevance of a study [15]. In this context, psychiatry is a field of particular interest. Psychiatrists are frequently criticized for their ties to the pharmaceutical industry and the impact of these ties has been intensely debated by top flight journals [16–18]. Pharmaceutical companies have been criticized for the high profits they received from antidepressants and antipsychotic sells. Their sells may have been influenced by substantial publication bias [13]. Furthermore, a controversy has emerged from reanalysis of data from sponsored study 329 that showed serious side effects of antidepressants which were not reported in the initial study [19]. Nevertheless, it is unknown whether these and other factors such as study outcomes and industry funding influence the scientific evaluation of an RCT. If psychiatrists are easily swayed by funding disclosure or positive outcomes, this could have direct impact on their prescribing behavior and influence their clinical decision making. Therefore, we aimed to assess how study outcome and industry funding disclosure influence psychiatrists’ perception of credibility and clinical relevance of results of a hypothetical RCT.

## Methods

### Abstract development

Three of the authors of this study extensively discussed the content of the proposed fictitious abstracts. We used the PubMed format (reproduced without the NCBI logo in Additional file 2: SF1–4) to make it appear like an original study. After producing a first draft of the abstracts by three authors, it was sent as a pilot version to 5 other psychiatrists to receive feedback on formulation, design, credibility and face-validity. This feedback resulted in minor modifications and thus the final version of the abstract. Each participant received the survey in Dutch containing an abstract in the English language describing an RCT for a non-existing novel antipsychotic drug ‘vinquerine’ in a fictitious journal (“the ArXX CXXX PsychXXX”). We chose to display the abstract in English since Dutch psychiatrists are comfortable in reading scientific literature in English. Four different abstracts were created based on reported study outcome (positive (a statistical significant difference compared with olanzapine) vs negative (no statistical differences compared with olanzapine)) and industry funding disclosure (yes/no). The original fake abstracts are included in (Additional file 2: Figures SF1–4). The fictional study compared the effects of the antipsychotic medication vinquerine to those of olanzapine and placebo on psychotic symptoms in patients with a first-episode psychosis. We chose olanzapine because it is an established and often used treatment for psychosis. Severe side effects were recorded in the fictitious abstract since these influence prescription behavior; the presence or absence of side effects often makes significant influence on the choices clinicians make in the treatment of psychiatric patients.

A positive outcome was reported by showing a statistically and clinically significant effect of vinquerine compared to olanzapine (an often applied first line treatment for psychosis) and placebo. In the positive outcome abstract, vinquerine was reported to have very limited side effects.

A negative outcome was reported as vinquerine and olanzapine being equally effective, but vinquerine showing important side effects. Both vinquerine and olanzapine were reported as being superior to placebo.

In the industry funded version, the second author was reported to be a consultant for a fictional pharmaceutical company (‘Olevy Pharmaceuticals’). We chose to put the conflict of interest (CoI) under the second author, rather than the first, since it was considered to be more consistent with existing scientific reporting. At the bottom of the fictitious industry funded abstracts, it was clearly stated that ‘this study was funded by a research grant from Olevy Pharmaceuticals’.

Identical methodological limitations were deliberately introduced in all versions of the abstract to make it consistent with Pubmed abstracts that, according to the authors, were judged to be average quality. We felt that reporting methods of average quality would make it more likely that the article would be scrutinized for validity. In contrast, a study without noticeable limitations could be received by clinicians as being fabricated or lacking veracity. The limitations of the fictitious abstract included a relatively small sample size (*n* = 303), unclear selection of study participants, non-blinded study design, a relatively short follow-up period (4-weeks), and the exclusion of non-compliant patients in a per-protocol analysis. The abstract-only approach was deliberately chosen since physicians frequently only read the abstract of potentially interesting scientific studies [20].

Statement: To prevent a potential confusion, we would like to underline that the abstracts were invented.

### Survey sample and randomization

VanDerHoef&Partners provided access to Dutch psychiatrists but were not involved in any part of the study (concept, design, analysis, and writing). One thousand five hundred sixty-six Dutch psychiatrists were randomized to receive an e-mail with a link to one of the following four abstracts: 1) negative outcome/industry funding disclosure, 2) negative outcome/no industry funding disclosure, 3) positive outcome/industry funding disclosure, or 4) positive outcome/no industry funding disclosure. The invitational e-mail and subsequent reminders were sent in May and June 2014 and included a brief statement to describe the goal of the study in very general terms (‘to determine how psychiatrists evaluate scientific research’). Participants were left unaware of the true design and intention of the study. The e-mail included a link to the online survey and the instructions on how-to opt-out of this study if they so chose. Two reminders were sent within a four week time frame to psychiatrists who did not respond to the initial email. If psychiatrists declined to respond, they were asked to follow an electronic link to a very brief online questionnaire to disclose their reasons for non-participation. Consent to participate was implied by following the link to the survey and completing the survey.

### Survey characteristics and outcomes

Each abstract was accompanied by a short questionnaire with 10 statements to determine the credibility (“How would you rate the credibility of the abstract?”), clinical relevance, methodological rigor (seven statements), and interest in reading the full article using a 10-points Likert scale (1: very poor, 10: extremely good). A control question (“please enter the score 3 for this question”) was included to check if participants did not randomly complete the survey. Methodological rigor was assessed with seven items, addressing different methodological characteristics: study design, methodology, statistical analysis, sample size, outcome measures, completeness of reporting and overall study quality [see Additional file 1: Table S1]. An equal-weighted sum score for the 7 items was calculated. To prevent that the order of questions have an undue influence on the answers respondents provided, the primary outcome questions were randomly distributed among the 10 questions.

After completion of the survey, participants’ attitudes towards the pharmaceutical industry were assessed. We also asked for financial ties with the pharmaceutical industry. Participants were asked if they had received a representative of a pharmaceutical company or if they had received any industry funding in the past 6 months. In addition, they were provided with four statements regarding the influence of industry funding on scientific clinical studies. Participants were asked to agree or disagree. At this stage of the survey, participants were not able to alter any of their previous answers.

Participant characteristics were obtained by asking respondents to report their gender, age, professional affiliations (academia, general hospital, or mental health care facility) and whether or not they had obtained a PhD. In addition, they were asked their place of residency, and whether the participant was actively involved in scientific research. For the complete survey with all the questions, see Additional file 3.

### Statistical analysis

Primary outcome measures were the perceived credibility and the judgement regarding clinical relevance of the study results reported in the fictitious abstract. Secondary outcomes were the level of interest in reading the full article, and global assessment of methodological rigor (sum score). All primary and secondary outcomes were scored on a 10-point Likert scale. We checked for distribution patterns of the primary and secondary outcomes and concluded that the distribution allows the use of ANOVA (see Additional file 1: Table S4). The survey software was constructed in a manner that prevented participants from submitting the survey unless all questions were completed. This was done to minimize missing data. First, possible interaction between the industry funding disclosure and study outcome was addressed. For the primary outcomes, the effect of funding disclosure and study outcome were analyzed using 2 × 2 ANOVA. Possible confounders and effect modifiers were analyzed, including participants’ self-reported attitude towards industry funding, and active relationships with the pharmaceutical industry. As a secondary, exploratory analysis, linear univariate and multivariate regression analyses were used to identify other possible determinants of perceived credibility and clinical relevance, including demographic and job specific factors, and active relations with industry. The Statistical Package for the Social Sciences (SPSS) statistics (Chicago USA 2011, version 20) was used for all statistical analyses.

## Results

### Participant characteristics

A total of 880 psychiatrists opened the invitation by email, to which 580 psychiatrists responded (66%), and 395 (45%) completed the full survey. Two respondents were excluded because they failed to correctly answer the control question. Demographic data and characteristics of the participants are presented in Table 1. Attitudes towards industry funding are included in Additional file 1: Tables S2 and S3).

**Table 1:** Demographic and professional characteristics of the participants

		*N* = 395	%
Gender	Male (%)	214	54
Age	Mean (range)	50 (27–72)	
Professional affiliation	Academia	40	10
	General Hospital	37	9
	Mental Health Institution	248	63
	Private Practice	63	16
	Other	38	10
Residency background	Academic Hospital	158	40
	General Hospital	4	1
	Mental Health Institution	200	51
	Other	32	8
Sub-specialty	Adult psychiatry	241	61
	Child psychiatry	90	23
	Geriatric psychiatry	25	6
	Other	37	9
Received representative of a pharm. Company	Yes	180	46
Received pharmacological funding	Yes	14	4
Scientifically active	Yes	131	33
PhD degree	Yes	104	27
Years employed as psychiatrist (range)		14 (0–40)	

### Industry funding disclosure and study outcome

#### Interaction analysis

No significant interaction was found between study outcome in the abstract and mentioning of the industry funding disclosure, both on the primary outcome credibility (*p* = 0.99) and the outcome clinical relevance (*p* = 0.41).

#### Industry funding disclosure does not affect credibility or relevance assessments

Industry funding disclosure was not significantly associated with perceived credibility (MD 0.12; 95% CI − 0.28 to 0.47) nor clinical relevance (MD 0.14; 95% CI − 0.54 to 0.27). Likewise, no significant effects were found for industry funding disclosure on the secondary outcomes ‘assessment of methodological rigor’ (MD 0.22; 95% CI − 1.82 to 2.17), and ‘interest in reading the full article’ (MD 0.14; 95% CI − 0.40 to 0.71) (Table 2).

**Table 2:** ANOVA analysis of the primary and secondary outcomes of the abstract with or without funding disclosure and with a positive or negative study outcome

	Funding (*N* = 206)	No Funding (*N* = 187)	Mean Difference (MD)	95% CI
Credibility^a^	4.64	4.76	0.12	−0.28 to 0.47
Clinical relevance	5.30	5.16	0.14	−0.54 to 0.27
Interest in reading the full article	4.51	4.65	0.14	−0.40 to 0.71
Methodological quality (sum score)^b^	38.65	38.87	0.22	−1.82 to 2.17
	Negative outcome (*N* = 200)	Positive outcome (*N* = 193)	Mean Difference (MD)	95% CI
Credibility^a^	**5.10**	**4.29**	**0.81**	**0.43 to 1.18**
Clinical relevance^a^	5.16	5.30	0.14	−0.28 to 0.53
Interest in reading the full article^a^	**4.31**	**4.85**	**0.54**	**0.09 to 1.12**
Methodological quality (sum score)^b^	5,63	5,44	0.19	−3.31 to 0.68

^a^10-point Likertscale score

^b^Average score on a 10-point Likertscale of the seven individual items regarding methodological quality (see Additional file 1: Table S1)

#### Positive study outcomes are perceived as less credible

A positive study outcome of the fictional RCT was associated with significantly lower scores on credibility (MD 0.81; 95% CI 0.43 to 1.18) but not ‘clinical relevance’ (MD 0.14; 95% CI − 0.28 to 0.53), compared to a negative study outcome. The secondary outcome ‘interest in reading the full article’ (MD 0.54, 95% CI 0.09 to 1.12) was statistically significant. The other outcome ‘assessment of methodological rigor’ (MD 0.19; 95% CI − 3.31 to 0.68) was not significantly influenced by the reported study outcome (Table 2).

#### No effect modification by relations with or attitude towards industry funding

We investigated whether participants’ active relations with- and attitude towards- industry were effect modifiers or confounders in the observed relations between study outcome and the primary outcomes. Both for perceived credibility and clinical relevance, no interactions or confounding were found for pharmaceutical industry relations and attitude towards industry funding (data not shown). Nevertheless, psychiatrists were well aware of possible industry effects, with an average score of 8.0 of 10 for the statement that “*a pharmaceutical company can influence study results”*, a score of 7.4 out of 10 for the statement that “f*unding has an effect on the quality of research*”, and 6.9 out of 10 for the statement that “*funding from a pharmaceutical company has a negative influence on the validity of research results*” (see Additional file 1: Table S3).

### Professional characteristics that influence perceived credibility and clinical relevance

In a secondary exploratory analysis, we investigated whether professional characteristics affect perceived credibility and clinical relevance scores in the total study population. Credibility of the RCT was negatively associated with having scientific experience (e.g. having a PhD or being scientifically active) (MD -0.6; 95% CI − 0.99 to − 0.21), and positively associated with having recently received a pharmaceutical representative (MD 0.63; 95% CI 0.25 to 1.01), funding from a pharmaceutical company (MD 1.27; 95% CI 0.25 to 2.30) or being employed in a general mental health institution (MD 0.46; CI 0,06 to 0,85). Multivariate analyses of these variables did not affect any of these results (data not shown).

Psychiatrists interpreted clinical relevance to be lower if they had scientific experience (‘having a PhD’ or ‘being scientifically active’) (MD -0.43; 95% CI − 0.85 to − 0.02), were longer active as a psychiatrist (beta − 0.02; 95% CI − 0.04 to 0.00) and had received residency training in a general mental health institution rather than an academic hospital (MD 0.40; 95% CI − 0.80 to 0.00). Multivariate analysis only showed significant associations for scientific experience and residency training in a general non-academic mental health institution (data not shown).

## Discussion

This study demonstrates that psychiatrists have more confidence in the validity of a negative outcome than a positive outcome of a fictitious study assessing a novel antipsychotic drug. In apparent contrast, we did not find a difference in the evaluation of a scientific abstract when participants were displayed an industry funding disclosure with the fictitious abstract. The reader’s attitudes towards or relationships with the pharmaceutical industry did not influence this analysis.

The relative distrust of a positive study outcome in our study is in agreement with the recognition that the medical literature suffers from positive outcome bias [6, 7], particularly in drug studies [21, 22]. Positive outcome bias has also been convincingly demonstrated in psychiatric literature, for example on the effectiveness of antidepressants [4, 23] and antipsychotics [24]. Presumably, respondents were aware of this bias, generating hesitation to accept results that may be considered ‘too good to be true’. The fact that positive outcomes are more critically appraised by psychiatrists is consistent with another study that highlights the influence of positive outcomes in paper acceptance rates in peer review [2]. These results are not fully comparable as peer reviewers have other criteria to assess a scientific paper than clinicians.

Although the effects of industry funding on study outcomes has been well-established both in the general medical literature [5] and in the psychiatric literature [11], no effect was found for funding disclosure on the credibility or perceived relevance of, in particular the positive outcome version of the abstract. Moreover, participants’ attitudes towards pharmaceutical industry funding did not influence participants’ appraisal of the fictitious abstract. (see Additional file 1: Table S3). Participants with overall high scores on statements that pharmaceutical companies can unduly influence study results were no more likely to question the validity of the fictitious abstract, even though the abstract clearly disclosed the funding. We cannot infer why these attitudes did not affect the results of our study. Apparently, psychiatrists did not automatically connect their perception of funding effects to the actual interpretation of the abstract we sent them. It may well be that respondents account for the mentioned disclosures, and may think that their knowledge will not influence the perception of research results.

Disclosure of industry funding in itself does not make study results more or less valid [25]. Future research should investigate whether funding disclosures on scientific abstracts are warranted. One might argue that reporting industry disclosures could unjustifiable influence the perceived credibility of research results simply because it was funded by or carried out by the pharmaceutical industry.

The recent media attention on study 329 regarding antidepressant drugs reinforces the need for individual patient data and original study protocols to increase the validity of scientific results as registration of clinical trials is insufficient [19].

Participants were a random sample from ±3500 Dutch psychiatrists. Nevertheless, around 27% of the participants had a PhD degree, which is higher than the average among community psychiatrists. Probably, psychiatrists with a PhD are more likely to participate in our survey, as they would feel more competent to assess a scientific abstract. Moreover, in light of the response rate of 45%, we checked whether the study results are generalizable to the total population of Dutch psychiatrists. With 54% of the survey sample being male compared to 59% in another recent study of Dutch psychiatrists, [26], there is no reason to assume that the results are not generalizable.

Secondary analyses showed that credibility scores were higher in psychiatrists with active relations with pharmaceutical companies and this was independent of industry funding disclosure. Credibility scores of the fictitious abstract were lower for the psychiatrists with scientific research experience. These results suggest that participants’ relationships with the pharmaceutical industry can influence study appraisal in a more positive or less critical manner.

To our knowledge, this is the first randomized study to investigate whether industry funding disclosure and study outcomes can influence the opinion of psychiatrists on the credibility and relevance of clinical research. In contrast, a somewhat smaller, previous study among 269 internists suggested that industry funding disclosure resulted in lower scores on methodological rigor and less confidence in the results [15]. The study design with different methodological approaches and the inclusion of three hypothetical drugs may have played a role in the discrepancy between these two studies. Additionally, it may be possible that internists are more critical towards pharmaceutical industry funded studies compared to psychiatrists.

The results of this study should be cautiously interpreted in light of several limitations. First, internet-based questionnaires can suffer from a selective response bias, predominantly attracting participants who feel capable of assessing a scientific abstract. In our study, the response rate of 45% was relatively high compared to other internet-based surveys for physicians [27, 28]. To calculate the response rate, we used the psychiatrists who opened the email as the denominator. A stricter calculation of the response rate would use all invitees (*n* = 1566) - whether they opened the email or not - in our response rate determination. This would have resulted in a response rate of 25% (395 out of 1566 invitations). We did not survey reasons for non-response. Most likely, psychiatrists lack time and energy to engage in online surveys. Non-response could also be due to the topic of the survey. Possibly, some psychiatrists might have been reluctant to participate as they could feel not competent enough to judge a scientific abstract on its quality.

Second, a 19% difference (Cohen’s d 0.43) in perceived credibility score and the 13% difference (Cohen’s d 0.19) in interest in reading the full article between the two groups is large enough to allow firm conclusions. Third, we did not find a significant difference between funding disclosure and perceived credibility. Although it may be enticing to conclude that there is no relation between the two variables, we cannot draw this conclusion from our data as no evidence of an association is not evidence of no association. Fourth, it might be that the participants perceived the positive study as invalid because olanzapine is a well-established reference antipsychotic drug. Furthermore, they might have perceived the negative study as more realistic in terms of symptom reduction. Fifth, the side effect profile was different in the positive outcome abstract compared to the negative outcome abstract. This was done intentionally to assure that the positive outcome abstract would be perceived as a positive study. However, this may have had a collateral effect on the perceived credibility. Reporting severe side effects might improve the intuitive credibility of an abstract as psychiatrists frequently discuss side effects with patients and are focused on side effects in their treatments.

Finally, the question remains if the responders did notice the presence of the disclosure of industry funding. Some respondents may not have noticed the disclosure of industry funding in the scientific abstract, even though all seven psychiatrists who pretested the fictitious abstract noticed it. Participants may also have assumed that the study on a novel antipsychotic would automatically be funded by industry even without an explicit disclosure statement.

## Conclusion

In this randomized study among psychiatrists, the effect of industry funding disclosure was not associated with the reliability and relevance of the results of a randomized clinical trial for a fictitious antipsychotic drug. In contrast, psychiatrists more critically interpreted the abstract of the RCT when a positive outcome was reported. Our results are timely in light of the recent discussion on the effects of industry-physician relations in several leading medical journals [16, 17, 29]. There is a striking discrepancy between psychiatrists’ attitudes towards the pharmaceutical industry and the effects that funding disclosure has on their perceived credibility and judgement of clinical relevance of the RCT.

Nevertheless, the credibility of the RCT was lower in psychiatrists with more scientific experience and higher when psychiatrists received a pharmaceutical representative or received funding from a pharmaceutical company. Future research could investigate whether explicit funding disclosures on scientific abstracts in databases such as warranted.

## Additional files

Additional file 1: **Table S1.** Perceived methodological rigor Average scores of individuals statements regarding the methodological rigor of the abstract. **Table S2.** Attitudes towards pharmaceutical funding related to credibility and clinical relevance Univariate regression analysis examining whether attitudes towards industry funding affects the perceived credibility and clinical relevance. Regression coefficients and 95% confidence intervals (CIs) are shown. **Table S3.** Attitude towards pharmaceutical funding Mean scores of four statements regarding the attitude of participants towards pharmaceutical companies and their involvement in research.** Table S4.** Distribution of primary and secondary outcomes. (DOC 52 kb)

Additional file 2: **SF 1.** Pubmed style fake abstract with positive outcome and funding disclosure. **SF 2.** Pubmed style fake abstract with positive outcome and no funding disclosure. **SF 3.** Pubmed style fake abstract with negative outcome and funding disclosure **SF 4.** Pubmed style fake abstract with negative outcome and no funding disclosure. (PDF 893 kb)

Additional file 3. Survey_Questions. (The translation of the survey questions). (DOC 27 kb)

## Data Availability

An anonymized version of the dataset used is available from the corresponding author on reasonable request.

## Abbreviations

ANOVA: Analysis of Variance
CI: Confidence Interval
CoI: Conflict of Interest
MD: Mean Difference
NCBI: National Center for Biotechnology Information
RCT: Randomized Controlled Trial
SPSS: Statistical Package for the Social Sciences
