# Supplementary material for: The effects of industry funding and positive outcomes in the interpretation of clinical trial results: a randomized trial among Dutch psychiatrists
Source: BMC Med Ethics. 2019 Sep 18;20:64. doi: 10.1186/s12910-019-0405-7 (PMC6749641; doi:10.1186/s12910-019-0405-7)
Supplement: Supplementary file 1 — Table S1. Perceived methodological rigor Average scores of individuals statements regarding the methodological rigor of the abstract. Table S2. Attitudes towards pharmaceutical funding related to credibility and clinical relevance Univariate regression analysis examining whether attitudes towards industry funding affects the perceived credibility and clinical relevance. Regression coefficients and 95% confidence intervals (CIs) are shown. Table S3. Attitude towards pharmaceutical funding Mean scores of four statements regarding the attitude of participants towards pharmaceutical companies and their involvement in research. Table S4. Distribution of primary and secondary outcomes. (DOC 52 kb) [file 12910_2019_405_MOESM1_ESM.doc]

**SUPPLEMENTARY MATERIAL**

***Supplementary Table S1. Perceived methodological rigor*** *Average scores of individuals statements regarding the methodological rigor of the abstract.*

|  | Funding disclosure | No funding disclosure | Positive outcome | Negative outcome |
| --- | --- | --- | --- | --- |
| **Study design** | 5.7 | 5.6 | 5.7 | 5.6 |
| **Methodology** | 5.7 | 5.7 | 5.9 | 5.5 |
| **Statistical analysis** | 5.2 | 5.1 | 5.3 | 5.0 |
| **Sample size** | 6.6 | 6.6 | 6.5 | 6.7 |
| **Outcome measures** | 5.9 | 6.1 | 6.0 | 5.9 |
| **Completeness of reporting** | 4.5 | 4.6 | 4.7 | 4.3 |
| **Overall study quality** | 5.2 | 5.2 | 5.3 | 5.1 |

***Supplementary table S2. Attitudes towards pharmaceutical funding related to credibility and clinical relevance*** *Univariate regression analysis examining whether attitudes towards industry funding affects the perceived credibility and clinical relevance. Regression coefficients and 95% confidence intervals (CIs) are shown.*

|  | **Credibility** | | **Clinical relevance** | |
| --- | --- | --- | --- | --- |
|  | **Beta** | **95% CI** | **Beta** | **95% CI** |
| A pharmaceutical company can influence study results | -0.11 | -0.22 to 0.001 | -0.03 | -0.14 to 0.09 |
| Funding has an effect on the quality of research | -0.03 | -0.13 to 0.82 | -0.09 | -0.20 to 0.03 |
| Funding from a pharmaceutical company has a negative influence on the validity of research results | -0.004 | -0.10 to 0.92 | 0.001 | -0.10 to 0.10 |
| If a scientist works for a pharmaceutical company, his/her study results are not trustworthy | -0.004 | -0.10 to 0.92 | 0.001 | -0.10 to 0.10 |

***Supplementary table S3.*** ***Attitude towards pharmaceutical funding*** *Mean scores of four statements (ranging 0-10) regarding the attitude of participants towards pharmaceutical companies and their involvement in research*

|  | **Negative study outcome** | | **Positive study outcome** | |
| --- | --- | --- | --- | --- |
|  | **Funding disclosure** | **No Funding disclosure** | **Funding disclosure** | **No Funding disclosure** |
| A pharmaceutical company can influence study results (average (SD)) | 8.2 (1.7) | 7.7 (1.9) | 8.1 (1.6) | 8.0(1.6) |
| Funding has an effect on the quality of research (average (SD)) | 7.7 (1.7) | 7.3 (1.8) | 7.2 (2.0) | 7.5(1.6) |
| Funding from a pharmaceutical company has a negative influence on the validity of research results (average (SD)) | 7.0 (1.9) | 6.5 (2.1) | 7.0 (2.0) | 7.0(1.7) |
| If a scientist works for a pharmaceutical company, his/her study results are not trustworthy (average (SD)) | 5.4 (1.9) | 5.4 (2.1) | 5.7 (2.1) | 5.8(2.0) |

**Supplementary table S4** **Distribution of primary and secondary outcomes**

|  | Mean | +/- SD | Skewness | Kurtosis |
| --- | --- | --- | --- | --- |
| Credibility | 4.70 | 1.93 | -0.018 | -0.759 |
| Clinical relevance | 5.23 | 2.02 | -0.213 | -0.661 |
| Interest in reading the full article | 4.58 | 2.79 | 0.288 | -1.022 |
| Methodological quality | 5.19 | 1.79 | -0.301 | -0.552 |
