# Supplementary material for: The effects of industry funding and positive outcomes in the interpretation of clinical trial results: a randomized trial among Dutch psychiatrists
Source: BMC Med Ethics. 2019 Sep 18;20:64. doi: 10.1186/s12910-019-0405-7 (PMC6749641; doi:10.1186/s12910-019-0405-7)
Supplement: Supplementary file 2 — SF 1. Pubmed style fake abstract with positive outcome and funding disclosure. SF 2. Pubmed style fake abstract with positive outcome and no funding disclosure. SF 3. Pubmed style fake abstract with negative outcome and funding disclosure SF 4. Pubmed style fake abstract with negative outcome and no funding disclosure. (PDF 893 kb) [file 12910_2019_405_MOESM2_ESM.pdf]

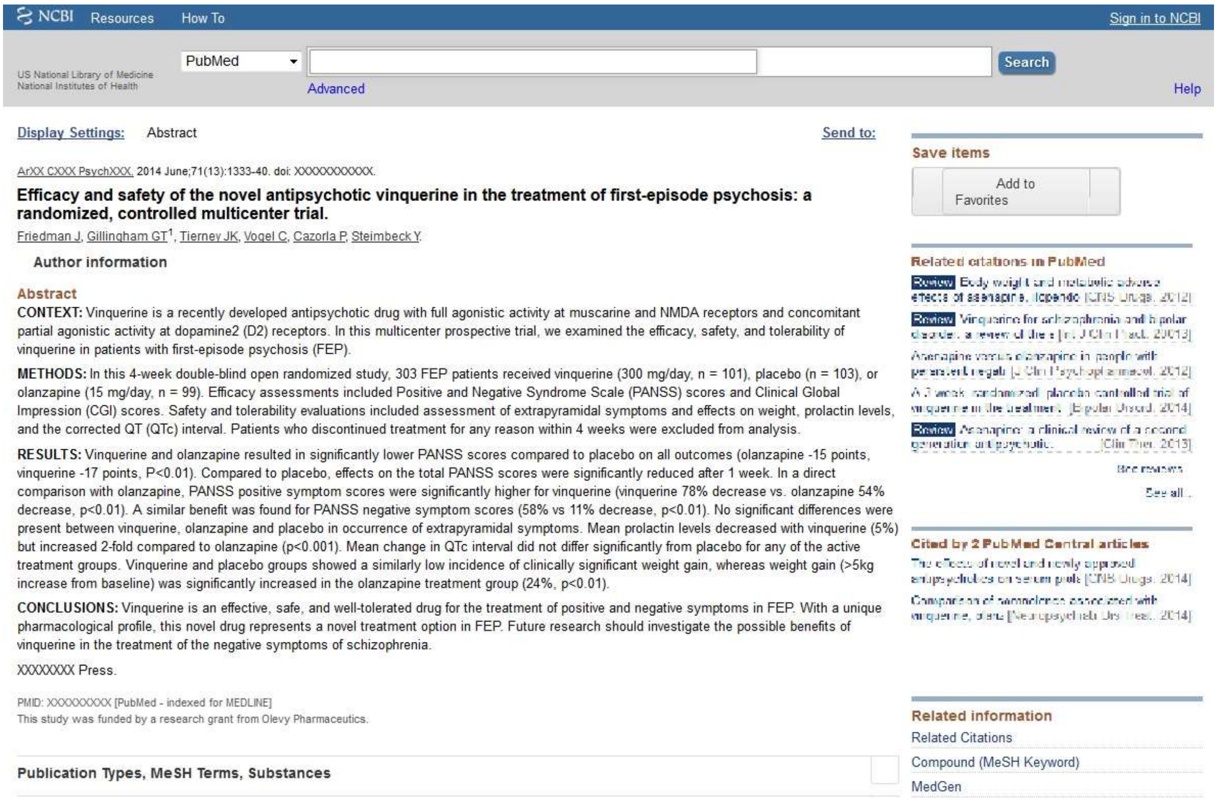

NCBI Resources How To Sign in to NCBI

US National Library of Medicine
National Institutes of Health

PubMed Search

Advanced Help

Display Settings: Abstract Send to:

ArXX CXXX PsychXXX. 2014 June;71(13):1333-40. doi: XXXXXXXXXXX.

Efficacy and safety of the novel antipsychotic vinquerine in the treatment of first-episode psychosis: a randomized, controlled multicenter trial.

Friedman J, Gillingham GT[1], Tierney JK, Vogel C, Cazorla P, Steimbeck Y.

Author information

Abstract

CONTEXT: Vinquerine is a recently developed antipsychotic drug with full agonistic activity at muscarine and NMDA receptors and concomitant partial agonistic activity at dopamine2 (D2) receptors. In this multicenter prospective trial, we examined the efficacy, safety, and tolerability of vinquerine in patients with first-episode psychosis (FEP).

METHODS: In this 4-week double-blind open randomized study, 303 FEP patients received vinquerine (300 mg/day, n = 101), placebo (n = 103), or olanzapine (15 mg/day, n = 99). Efficacy assessments included Positive and Negative Syndrome Scale (PANSS) scores and Clinical Global Impression (CGI) scores. Safety and tolerability evaluations included assessment of extrapyramidal symptoms and effects on weight, prolactin levels, and the corrected QT (QTc) interval. Patients who discontinued treatment for any reason within 4 weeks were excluded from analysis.

RESULTS: Vinquerine and olanzapine resulted in significantly lower PANSS scores compared to placebo on all outcomes (olanzapine -15 points, vinquerine -17 points, P<0.01). Compared to placebo, effects on the total PANSS scores were significantly reduced after 1 week. In a direct comparison with olanzapine, PANSS positive symptom scores were significantly higher for vinquerine (vinquerine 78% decrease vs. olanzapine 54% decrease, p<0.01). A similar benefit was found for PANSS negative symptom scores (58% vs 11% decrease, p<0.01). No significant differences were present between vinquerine, olanzapine and placebo in occurrence of extrapyramidal symptoms. Mean prolactin levels decreased with vinquerine (5%) but increased 2-fold compared to olanzapine (p<0.001). Mean change in QTc interval did not differ significantly from placebo for any of the active treatment groups. Vinquerine and placebo groups showed a similarly low incidence of clinically significant weight gain, whereas weight gain (>5kg increase from baseline) was significantly increased in the olanzapine treatment group (24%, p<0.01).

CONCLUSIONS: Vinquerine is an effective, safe, and well-tolerated drug for the treatment of positive and negative symptoms in FEP. With a unique pharmacological profile, this novel drug represents a novel treatment option in FEP. Future research should investigate the possible benefits of vinquerine in the treatment of the negative symptoms of schizophrenia.

XXXXXXXX Press.

PMID: XXXXXXXXX [PubMed - indexed for MEDLINE]

This study was funded by a research grant from Olevy Pharmaceutics.

Publication Types, MeSH Terms, Substances

Save items

Add to Favorites

Related citations in PubMed

See all...

Cited by 2 PubMed Central articles

Related information

Related Citations

Compound (MeSH Keyword)

MedGen

SF 1 Pubmed style fake abstract with positive outcome and funding disclosure

NB. the manuscript has been altered to remove references to journal title, pubmed ID, copy right, etc. where this information was the same as real world entities. In the original survey this information was unblurred to better mimic a PubMed abstract

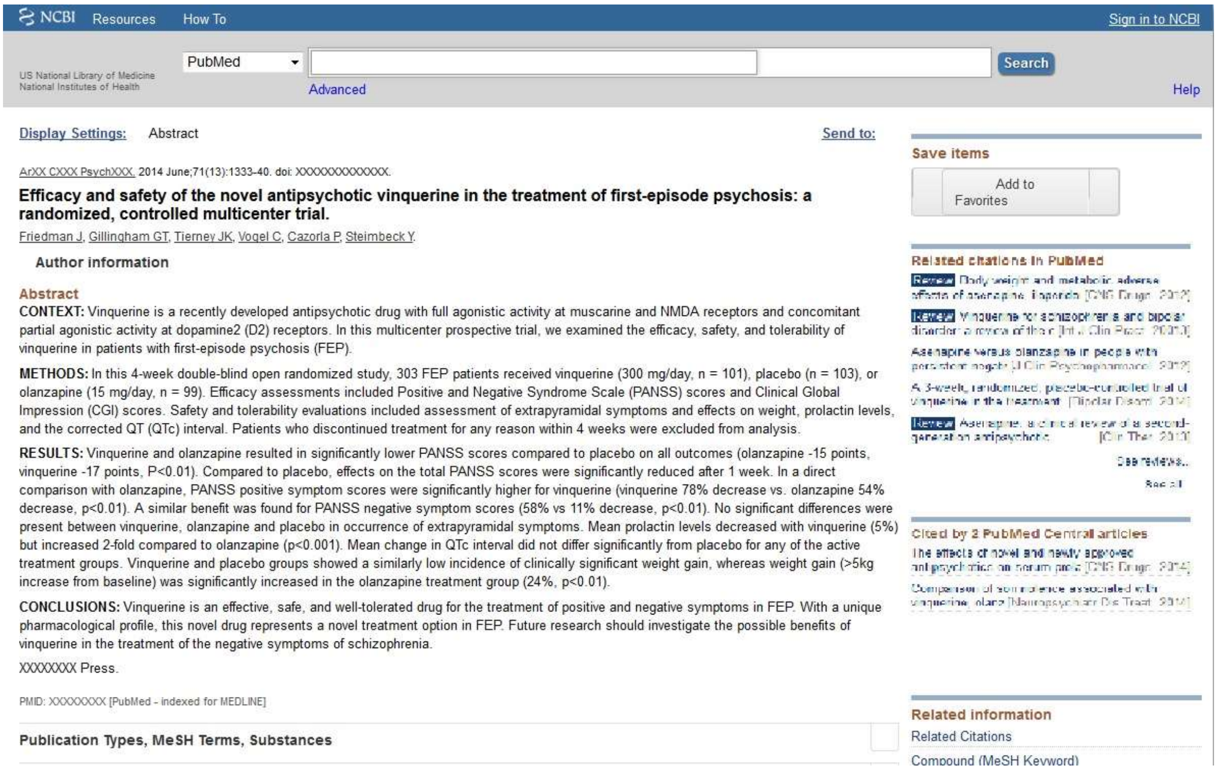

NCBI Resources How To Sign in to NCBI

US National Library of Medicine
National Institutes of Health

PubMed Search

Advanced Help

Display Settings: Abstract Send to:

ArXX CXXX PsychXXX. 2014 June;71(13):1333-40. doi: XXXXXXXXXXXXX.

Efficacy and safety of the novel antipsychotic vinquerine in the treatment of first-episode psychosis: a randomized, controlled multicenter trial.

Friedman J, Gillingham GT, Tierney JK, Vogel C, Cazorla P, Steimbeck Y.

Author information

Abstract

CONTEXT: Vinquerine is a recently developed antipsychotic drug with full agonistic activity at muscarine and NMDA receptors and concomitant partial agonistic activity at dopamine2 (D2) receptors. In this multicenter prospective trial, we examined the efficacy, safety, and tolerability of vinquerine in patients with first-episode psychosis (FEP).

METHODS: In this 4-week double-blind open randomized study, 303 FEP patients received vinquerine (300 mg/day, n = 101), placebo (n = 103), or olanzapine (15 mg/day, n = 99). Efficacy assessments included Positive and Negative Syndrome Scale (PANSS) scores and Clinical Global Impression (CGI) scores. Safety and tolerability evaluations included assessment of extrapyramidal symptoms and effects on weight, prolactin levels, and the corrected QT (QTc) interval. Patients who discontinued treatment for any reason within 4 weeks were excluded from analysis.

RESULTS: Vinquerine and olanzapine resulted in significantly lower PANSS scores compared to placebo on all outcomes (olanzapine -15 points, vinquerine -17 points, P<0.01). Compared to placebo, effects on the total PANSS scores were significantly reduced after 1 week. In a direct comparison with olanzapine, PANSS positive symptom scores were significantly higher for vinquerine (vinquerine 78% decrease vs. olanzapine 54% decrease, p<0.01). A similar benefit was found for PANSS negative symptom scores (58% vs 11% decrease, p<0.01). No significant differences were present between vinquerine, olanzapine and placebo in occurrence of extrapyramidal symptoms. Mean prolactin levels decreased with vinquerine (5%) but increased 2-fold compared to olanzapine (p<0.001). Mean change in QTc interval did not differ significantly from placebo for any of the active treatment groups. Vinquerine and placebo groups showed a similarly low incidence of clinically significant weight gain, whereas weight gain (>5kg increase from baseline) was significantly increased in the olanzapine treatment group (24%, p<0.01).

CONCLUSIONS: Vinquerine is an effective, safe, and well-tolerated drug for the treatment of positive and negative symptoms in FEP. With a unique pharmacological profile, this novel drug represents a novel treatment option in FEP. Future research should investigate the possible benefits of vinquerine in the treatment of the negative symptoms of schizophrenia.

XXXXXXXX Press.

PMID: XXXXXXXXX [PubMed - indexed for MEDLINE]

Publication Types, MeSH Terms, Substances

Save items

Add to Favorites

Related citations in PubMed

See reviews...

See all

Cited by 2 PubMed Central articles

Related information

Related Citations

Compound (MeSH Keyword)

SF 2 Pubmed style fake abstract with positive outcome and no funding disclosure

NB. the manuscript has been altered to remove references to journal title, pubmed ID, copy right, etc. where this information was the same as real world entities. In the original survey this information was unblurred to better mimic a PubMed abstract

ArXX CXXX PsychXXX. 2014 June;71(13):1333-40. doi: XXXXXXXXXXXXXXXXX.

Efficacy and safety of the novel antipsychotic vinquerine in the treatment of first-episode psychosis: a randomized, controlled multicenter trial.

Friedman J, Gillingham GT[1], Tierney JK, Vogel C, Cazorla P, Steimbeck Y.

Author information

Abstract

CONTEXT: Vinquerine is a recently developed antipsychotic drug with full agonistic activity at muscarine and NMDA receptors and concomitant partial agonistic activity at dopamine2 (D2) receptors. In this multicenter prospective trial, we examined the efficacy, safety, and tolerability of vinquerine in patients with first-episode psychosis (FEP).

METHODS: In this 4-week double-blind open randomized study, 303 FEP patients received vinquerine (300 mg/day, n = 101), placebo (n = 103), or olanzapine (15 mg/day, n = 99). Efficacy assessments included Positive and Negative Syndrome Scale (PANSS) scores and Clinical Global Impression (CGI) scores. Safety and tolerability evaluations included assessment of extrapyramidal symptoms and effects on weight, prolactin levels, and the corrected QT (QTc) interval. Patients who discontinued treatment for any reason within 4 weeks were excluded from analysis.

RESULTS: Vinquerine and olanzapine resulted in significantly lower PANSS scores compared to placebo on all outcomes (olanzapine: 15 points, vinquerine: 17 points, P<0.01). Compared to placebo, effects on the total PANSS scores were significantly reduced after 1 week. In a direct comparison with olanzapine, vinquerine PANSS positive symptom scores were slightly lower for vinquerine (vinquerine 48% decrease vs. olanzapine 54% decrease, p = 0,15). A similar result was found for PANSS negative symptom scores (18% vs 11% decrease , p=0.45). No significant differences were present between vinquerine, olanzapine and placebo in the occurrence of extrapyramidal symptoms. Mean prolactin levels increased 2-fold with vinquerine but increased slightly (5%) with olanzapine (p<0.001). Mean change in QTc interval did not differ significantly from placebo for any of the active treatment groups. Placebo groups showed a low incidence of clinically significant weight gain, whereas weight gain (>5kg increase from baseline) was significantly increased in both active treatment groups (14% for vinquerine and 17% for olanzapine, p<0.01).

CONCLUSIONS: In this 4-week follow up study, vinquerine appeared to be effective for the treatment of positive symptoms in FEP. Nevertheless, significant increases in prolactin levels and weight gain occurred, and no significant differences were found between olanzapine and vinquerine. Future research and larger studies with a longer follow up period should investigate the possible benefits of vinquerine in the treatment of schizophrenia.

XXXXXXXX Press

PMID: XXXXXXXX [PubMed - indexed for MEDLINE]
This study was funded by a research grant from Olevy Pharmaceutics.

Publication Types, MeSH Terms, Substances

Save items

Add to Favorites

Related citations in PubMed

Review Body weight and metabolic adverse effects of asenapine, iloperidone [CNS Drugs. 2012]

Review Vinquerine for schizophrenia and bipolar disorder: a review of the [illegible] [Int J Clin Pract. 2013]

Asenapine versus olanzapine in people with persistent negati [J Clin Psychopharmacol. 2012]

A 3-week, randomized, placebo-controlled trial of vinquerine in the treatment [Bipolar Disord. 2014]

Review Asenapine: a clinical review of a second-generation antipsychotic. [Clin Ther. 2012]

See reviews...

See all...

Cited by 2 PubMed Central articles

The effects of novel and newly approved antipsychotics on serum prola [CNS Drugs. 2014]

Comparison of somnolence associated with vinquerine, olanz [Neuropsychiatr Dis Treat. 2014]

Related information

Related Citations

Compound (MeSH Keyword)

MedGen

SF 3 Pubmed style fake abstract with negative outcome and funding disclosure

NB. the manuscript has been altered to remove references to journal title, pubmed ID, copy right, etc. where this information was the same as real world entities. In the original survey this information was unblurred to better mimic a PubMed abstract

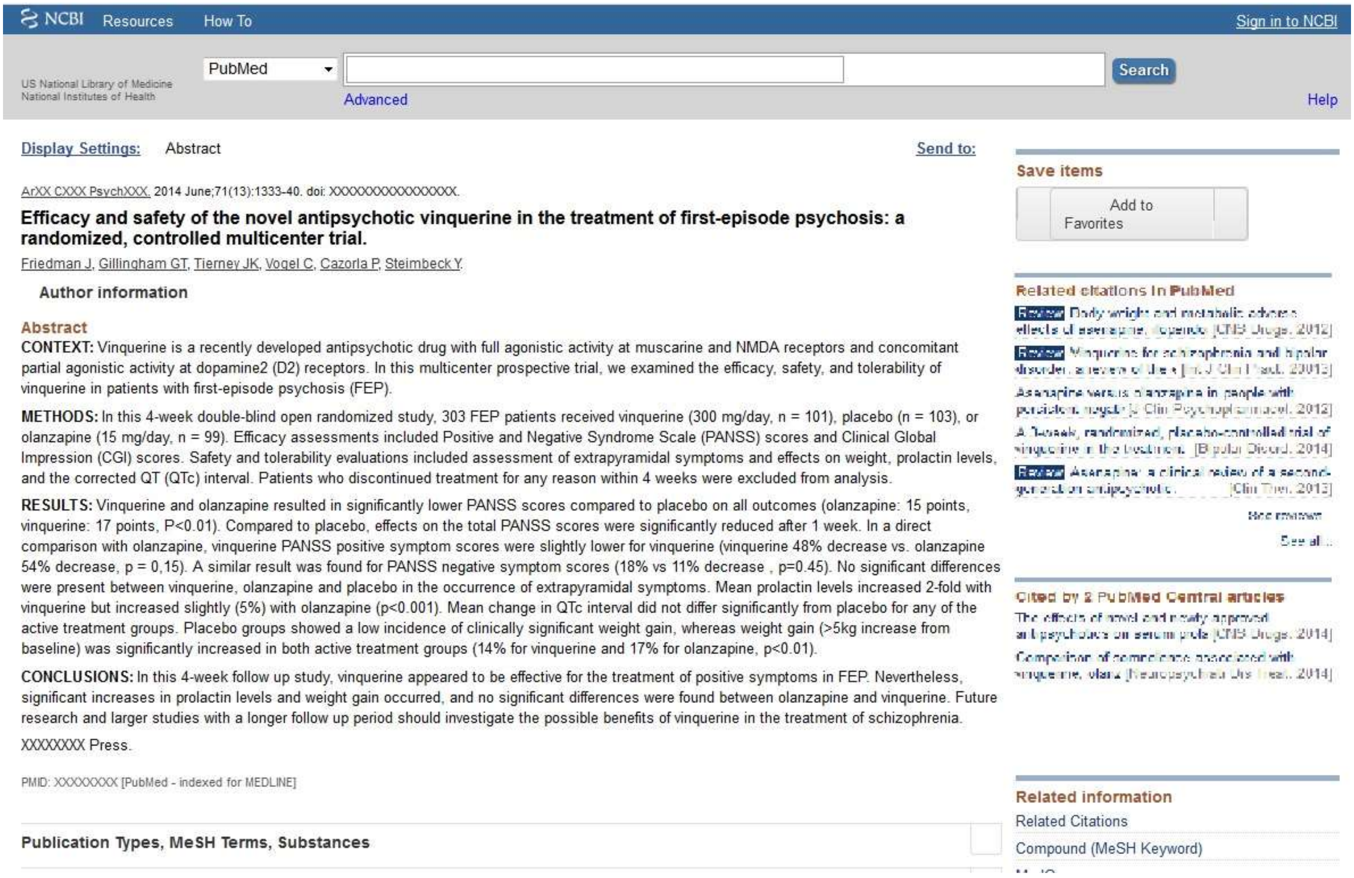

NCBI Resources How To Sign in to NCBI

US National Library of Medicine
National Institutes of Health

PubMed Search
Advanced Help

Display Settings: Abstract Send to:

ArXX CXXX PsychXXX. 2014 June;71(13):1333-40. doi: XXXXXXXXXXXXXXXXX.

Efficacy and safety of the novel antipsychotic vinquerine in the treatment of first-episode psychosis: a randomized, controlled multicenter trial.

Friedman J, Gillingham GT, Tierney JK, Vogel C, Cazorla P, Steimbeck Y.

Author information

Abstract

CONTEXT: Vinquerine is a recently developed antipsychotic drug with full agonistic activity at muscarine and NMDA receptors and concomitant partial agonistic activity at dopamine2 (D2) receptors. In this multicenter prospective trial, we examined the efficacy, safety, and tolerability of vinquerine in patients with first-episode psychosis (FEP).

METHODS: In this 4-week double-blind open randomized study, 303 FEP patients received vinquerine (300 mg/day, n = 101), placebo (n = 103), or olanzapine (15 mg/day, n = 99). Efficacy assessments included Positive and Negative Syndrome Scale (PANSS) scores and Clinical Global Impression (CGI) scores. Safety and tolerability evaluations included assessment of extrapyramidal symptoms and effects on weight, prolactin levels, and the corrected QT (QTc) interval. Patients who discontinued treatment for any reason within 4 weeks were excluded from analysis.

RESULTS: Vinquerine and olanzapine resulted in significantly lower PANSS scores compared to placebo on all outcomes (olanzapine: 15 points, vinquerine: 17 points, P<0.01). Compared to placebo, effects on the total PANSS scores were significantly reduced after 1 week. In a direct comparison with olanzapine, vinquerine PANSS positive symptom scores were slightly lower for vinquerine (vinquerine 48% decrease vs. olanzapine 54% decrease, p = 0,15). A similar result was found for PANSS negative symptom scores (18% vs 11% decrease , p=0.45). No significant differences were present between vinquerine, olanzapine and placebo in the occurrence of extrapyramidal symptoms. Mean prolactin levels increased 2-fold with vinquerine but increased slightly (5%) with olanzapine (p<0.001). Mean change in QTc interval did not differ significantly from placebo for any of the active treatment groups. Placebo groups showed a low incidence of clinically significant weight gain, whereas weight gain (>5kg increase from baseline) was significantly increased in both active treatment groups (14% for vinquerine and 17% for olanzapine, p<0.01).

CONCLUSIONS: In this 4-week follow up study, vinquerine appeared to be effective for the treatment of positive symptoms in FEP. Nevertheless, significant increases in prolactin levels and weight gain occurred, and no significant differences were found between olanzapine and vinquerine. Future research and larger studies with a longer follow up period should investigate the possible benefits of vinquerine in the treatment of schizophrenia.

XXXXXXXX Press.

PMID: XXXXXXXX [PubMed - indexed for MEDLINE]

Publication Types, MeSH Terms, Substances

Save items

Add to Favorites

Related citations in PubMed

Review Body weight and metabolic adverse effects of asenapine, iloperidone [CNS Drugs. 2012]

Review Vinquerine for schizophrenia and bipolar disorder: a review of the e [Int J Clin Pract. 2013]

Asenapine versus olanzapine in people with persistent negati [J Clin Psychopharmacol. 2012]

A 3-week, randomized, placebo-controlled trial of vinquerine in the treatment [Bipolar Disord. 2014]

Review Asenapine: a clinical review of a second-generation antipsychotic. [Clin Ther. 2013]

See reviews...

See all...

Cited by 2 PubMed Central articles

The effects of novel and newly approved antipsychotics on serum prola [CNS Drugs. 2014]

Comparison of somnolence associated with vinquerine, olanz [Neuropsychiatr Dis Treat. 2014]

Related information

Related Citations

Compound (MeSH Keyword)

SF 4 Pubmed style fake abstract with negative outcome and no funding disclosure

NB. the manuscript has been altered to remove references to journal title, pubmed ID, copy right, etc. where this information was the same as real world entities. In the original survey this information was unblurred to better mimic a PubMed abstract
