# Supplementary material for: The effects of industry funding and positive outcomes in the interpretation of clinical trial results: a randomized trial among Dutch psychiatrists
Source: BMC Med Ethics. 2019 Sep 18;20:64. doi: 10.1186/s12910-019-0405-7 (PMC6749641; doi:10.1186/s12910-019-0405-7)
Supplement: Supplementary file 3 — Survey_Questions. (The translation of the survey questions). (DOC 27 kb) [file 12910_2019_405_MOESM3_ESM.doc]

Questions:

Below you will find some questions referring to the abstract that is shown at the left part of your screen. It is an abstract of a new antipsychotic which has recently been published in a scientific journal (Impact Factor 8). We ask you to assess the quality of the research on the basis of this abstract by the following questions:

(1 = very bad, 10 = very good)

1. What do you think of the design of the study?
   1 2 3 4 5 6 7 8 9 10
2. How do you rate the methods used in the article?
   1 2 3 4 5 6 7 8 9 10
3. What do you think of the statistical analysis?
   1 2 3 4 5 6 7 8 9 10
4. What do you think of the sample size?
   1 2 3 4 5 6 7 8 9 10
5. What do you think of the outcome measures used?
   1 2 3 4 5 6 7 8 9 10
6. How complete is the study that is described in the abstract?
   1 2 3 4 5 6 7 8 9 10
7. This is a control question. Fill in the number three here.
   1 2 3 4 5 6 7 8 9 10
8. Assess the quality of the research by giving a note (1-7).
   1 2 3 4 5 6 7 8 9 10
9. How credible do you consider the conclusions drawn?
   1 2 3 4 5 6 7 8 9 10
10. Wat is the clinical relevance of the research?
    1 2 3 4 5 6 7 8 9 10
11. Would you like to read the whole article?
    1 2 3 4 5 6 7 8 9 10

Open questions:
- What is the best aspect of the study design?
....
- What is the most important shortcoming of the study design
...

Last part of the survey:

(Psychiatrists can not return to the first part of the survey and they cannot see the abstract while answering the next questions)

(1 = very bad, 10 = very good) or (1 = strongly disagree, 10 = strongly agree)

1. Do you think that a pharmaceutical company may affect the outcome of research?
1 2 3 4 5 6 7 8 9 10
2. Do you believe that sponsorship effects the quality of research?
1 2 3 4 5 6 7 8 9 10
3. Do you think (industry) funding has a negative impact on the credibility of research results?
1 2 3 4 5 6 7 8 9 10
4. If a scientist works for a pharmaceutical company, his research results are not reliable
1 2 3 4 5 6 7 8 9 10

Did you receive in the last 6 months a medical representative from a pharmaceutical company?
- Yes

- No

Did you receive in the last 6 months funding from a pharmaceutical company (for whatever reason)?
- Yes

- No

Demographic data:

1. What is your gender?

- Male

- Female

2. What is your age?

... (open answer)
3. Where do you currently work as a psychiatrist?

- Academic

- Peripheral

- Mental health institution

- Private practice

- Not Practicing / retired

- Other (multiple answers possible)
4. What percentage of your time do you spent on patient contact?

- 0-25%

- 25-50%

- 50-75%

- 75-100%
5. And how much on training (residents in psychiatry and medical students)?

- 0-25%

- 25-50%

- 50-75%

- 75-100%
6. Are you scientifically active (publishing scientific papers)?

- Yes

- No
7. Did you complete a doctorate/PhD?

- Yes

- No
8. How much time do you spend on research?

- 0-5%

- 5-25%

- 25-50%

- 50-75%

- 75-100%

9. Where did you complete your training as a psychiatrist?

- University Medical Center

- Mental health institution

- General Hospital
10. Where do you primarily work as a psychiatrist?

- UMC

- Mental health
- Private practice

- Forensic psychiatry

- General hospital/consultation/liaison

- I am a resident in psychiatry

- Other:
11. For how long have you been a psychiatrist?

... (open answer)
12. What is your subspecialty in psychiatry?

- Child and Adolescent Psychiatry

- Adult

- Old age psychiatry

Among the participants we raffle three iPads. Fill in your email address to participate:
E-mail:

The results will be processed anonymously and respondents cannot be tracked down as a unique user. In addition, your data will absolutely be kept confidential and not disclosed to third parties.
